# Hypothalamic–Pituitary Autoimmunity in Patients Treated with Anti-PD-1 and Anti-PD-L1 Antibodies

**DOI:** 10.3390/cancers13164036

**Published:** 2021-08-11

**Authors:** Giuseppe Bellastella, Carla Carbone, Lorenzo Scappaticcio, Paolo Cirillo, Teresa Troiani, Floriana Morgillo, Maria Teresa Vietri, Carminia Maria Della Corte, Vincenzo De Falco, Stefania Napolitano, Maria Ida Maiorino, Annamaria De Bellis, Katherine Esposito

**Affiliations:** 1 Unit of Endocrinology and Metabolic Diseases, Department of Advanced Medical and Surgical Sciences, University of Campania “Luigi Vanvitelli”, 80138 Naples, Italy; carla.carbone@unicampania.it (C.C.); lorenzo.scappaticcio@unicampania.it (L.S.); paolo.cirillo@unicampania.it (P.C.); mariaida.maiorino@unicampania.it (M.I.M.); annamaria.debellis@unicampania.it (A.D.B.); 2 Medical Oncology, Department of Precision Medicine, University of Campania “Luigi Vanvitelli”, 80138 Naples, Italy; teresa.troiani@unicampania.it (T.T.); floriana.morgillo@unicampania.it (F.M.); carminiamaria.dellacorte@unicampania.it (C.M.D.C.); vincenzo.defalco2@unicampania.it (V.D.F.); stefania.napolitano@unicampania.it (S.N.); 3 Unit of Clinical and Molecular Pathology, Department of Precision Medicine, University of Campania “Luigi Vanvitelli”, 80138 Naples, Italy; mariateresa.vietri@unicampania.it; 4 Diabetes Unit, Department of Advanced Medical and Surgical Sciences, University of Campania “Luigi Vanvitelli”, 80138 Naples, Italy; katherine.esposito@unicampania.it

**Keywords:** APA, anti-pituitary antibodies, AHA, anti-hypothalamus antibodies, pituitary autoimmunity, anti-PD-1, anti-PD-L1

## Abstract

**Simple Summary:**

The aim of this study is to search for APA and AHA and related pituitary dysfunction in patients treated with immunotherapy. APA and AHA could represent markers for early detection of patients at risk of developing pituitary deficiencies related to immune checkpoint inhibitors and undergoing closer follow-up. Furthermore, this study aims to evaluate the correlation between the presence of AHA and APA and the clinical response to checkpoint inhibitor therapy. However, further prospective studies will be needed to confirm our results.

**Abstract:**

Background: Autoimmune hypophysitis is a frequent immune-related adverse event (irAE) in cancer patients treated with immunecheckpoint inhibitors. Studies seeking anti-pituitary (APA) and anti-hypothalamus (AHA) antibodies in patients treated with anti-PD-1 and anti-PD-L1 are scarce. The aim of this study is to search for APA and AHA and related pituitary dysfunction in patients treated with these agents. Methods:Cross-sectional and preliminary longitudinal studies were conducted at the Medical Oncology Unit and Endocrinology and Metabolic Diseases Unit of the University of Campania “Luigi Vanvitelli”. Fifty-four cancer patients on treatments with anti-PD-1 or anti-PD-L1 (Group 1) and 50 healthy controls were enrolled for a cross-sectional study; 13 cancer patients (Group 2) were enrolled for our preliminary longitudinal study. APA/AHA titers and changes in biochemical and hormonal profile were evaluated in Group 1; in Group 2, they were evaluated before and after nine weeks from the start of immunotherapy. Results: Patients of Group 1 showed a higher prevalence of APA and AHA than controls: 21 of them had APA, 16 had AHA, and 11 had both autoantibodies. In total, 7 of 13 patients in Group 2 became APA-positive and 3 became AHA-positive after nine weeks of immunotherapy, showing an increase in prolactin and a decrease in ACTH and IGF-1 levels compared with basal values. Conclusions:Anti-pituitary and anti-hypothalamus antibodies seem to play a pivotal role in hypothalamic–pituitary autoimmunity and secondary endocrine-related alterations evoked by anti-PD-1 and PD-L1 antibodies.

## 1. Introduction

Immune checkpoint inhibitors (ICIs) have been recently introduced as an effective and innovative therapy for the treatment of advanced cancer. ICIs are monoclonal antibodies that inhibit negative regulatory components of the immune response, thus stimulating T-cells to counteract cancer cells. Three specific targets of these pharmacological agents are the cytotoxic T-lymphocyte-associated antigen 4 (CTLA-4), the programmed cell death protein-1 (PD-1), and its ligands (PD-L1/PD-L2) [1,2]. The first ICI that received approval by the Food and Drug Administration in the United States for use in advanced-stage melanoma was the CTLA-4 inhibitor, ipilimumab. Subsequently, PD-1 inhibitors (nivolumab, pembrolizumab, cemiplimab) and PD-L1 inhibitors (atezolizumab, avelumab, durvalumab) were approved, and the spectrum of indications was widely extended to include melanoma, non-small-cell lung cancer (NSCLC), renal cell carcinoma, Hodgkin’s lymphoma, head and neck squamous cell cancer, and hepatocellular carcinoma [2]. Several autoimmune side effects from these agents have been described (termed immune-related adverse events (irAEs), and they can include multiple autoimmune endocrine diseases [3,4,5,6,7,8,9,10,11,12,13,14,15,16,17]. In particular, among these, an immune-targeted cancer therapy-related hypophysitis has been recognized as an endocrine irAE with a higher prevalence in patients treated with anti-CTLA-4 with respect to those treated with anti-PD and anti-PD-L1 agents [7,8,9,10,11,18]. However, studies investigating the occurrence of hypothalamic–pituitary autoimmunity in patients treated with PD-1 and PD-L1 inhibitors are scarce, and only anti-pituitary antibodies (APA) were detected in affected patients [19]. Since APA and also anti-hypothalamus (AHA) antibodies have been detected in patients with autoimmune hypophysitis, suggesting that an immune hypothalamic involvement may contribute to pituitary dysfunction [20,21], we investigated the possible occurrence of APA and AHA and related pituitary and satellite gland dysfunctions in patients treated with these agents.

## 2. Materials and Methods

### 2.1. Cross-Sectional Study Population

Fifty-four cancer patients (35 M, 19 F, aged 65 ± 10.7 years, Group 1) with advanced lung cancer or melanoma, referred to the Medical Oncology Unit of the University Hospital, University of Campania “Luigi Vanvitelli”, along a time span ranging from November 2019 to June 2020, on treatments with antibodies anti-PD-1 (nivolumab and pembrolizumab: 37 of them, Group 1a) or anti-PD-L1 (avelumab, atezolizumab, and durvalumab: 17 of them, Group 1b) for at least 9 weeks, were enrolled in the cross-sectional study together with 50 healthy adult controls. Nine weeks was the minimum considered period of treatment to be enrolled in the cross-sectional study since some ICIs are administered every three weeks, with irAEsalready reported after six weeks.The control group (23 M, 27 F) was recruited from healthy sex- and age-matched adults without other known causes of pituitary dysfunction nor individual or family history of autoimmune diseases and without prior treatment with immune checkpoint inhibitors.

### 2.2. Longitudinal Study Population

A two-year longitudinal study was planned, starting from June 2020: 13 newly diagnosed cancer patients (10 M, 3 F, aged 70.6 ± 10.4 years, Group 2) eligible for immunotherapy for advanced lung cancer or melanoma and starting treatment with anti-PD-1 (nivolumab and pembrolizumab: 7 patients), or anti-PD-L1 (avelumab, atezolizumab, and durvalumab: 6 patients) were enrolled in our longitudinal study before starting immunotherapy. Patients were and will be periodically studied for autoimmune, biochemical, and hormonal findings until the completion of the two years of observation. Here, we report and discuss the preliminary data obtained at the baseline observation and after nine weeks from the start of immunotherapy. In the longitudinal study, patients will be evaluated every two months for two years. The first time point was delayed by a week due to the COVID-19 pandemic.

### 2.3. Blood Test Evaluation

After giving their consent, all patients and controls involved in the cross-sectional and longitudinal studies had a venous blood sample taken for evaluation of glycemia, sodium, ACTH, cortisol, TSH, FT3, FT4, TgAb, TPOAb, LH, FSH, PRL, IGF1, and for determining the presence of APA and AHA. In particular, we sought these antibodies and changes in biochemical and basal hormonal profile in all patients in Group 1 (after a minimum of nine weeks of immunotherapy) and in controls and in the 13 patients in Group 2 before and after nine weeks of immunotherapy. Both studies were approved by the Ethics Committee of the University of Campania “Luigi Vanvitelli”—A.O.R.N. “OspedaledeiColli”.

### 2.4. APA and AHA Evaluation

All patients were tested for APA and AHA by a simple indirect immunofluorescence method (Immunoendocrinology Laboratory of the Endocrinology and Metabolic Diseases Unit of University of Campania “Luigi Vanvitelli”) on cryostat sections of young baboon pituitary gland and young baboon hypothalamus supplied by Halifax spa (Polverara, Pordenone, Italy) and Biomedissrl (Rome, Italy), respectively, as previously described [22,23,24]. In particular, unfixed cryostat sections of young normal baboon pituitary and hypothalamus were initially incubated with the sera. Then, serum samples were subsequently tested with fluorescein isothiocyanate-conjugated goat antihuman Ig to detect the presence of APA and AHA.

**Figure 1:**
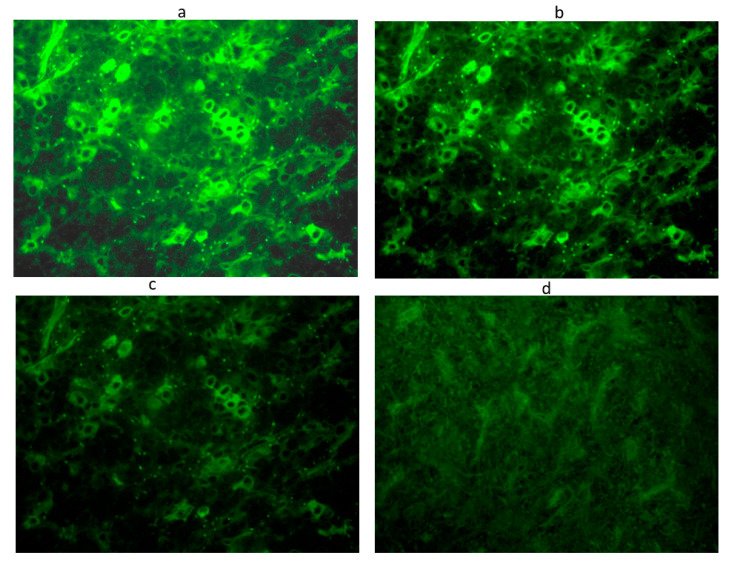
Anti-pituitary antibodies detected by immunofluorescence method in a serum positive at 1:32 titer: Intracytoplasmatic immunofluorescence of pituitary cells at different dilutions: 1:8 (**a**), 1:16 (**b**), 1:32 (**c**), until the disappearance of significant immunofluorescence at adilution of 1:64 (**d**). Scale bar: 40×.

**Figure 2:**
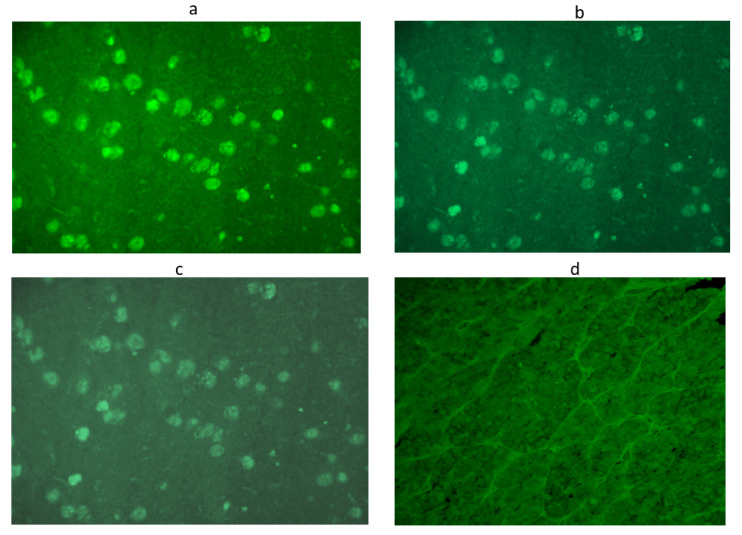
Anti-hypothalamus antibodies detected by immunofluorescence method in a serum positive at 1:32 titer: Intracytoplasmatic immunofluorescence of hypothalamic cells at different dilutions: 1:8 (**a**), 1:16 (**b**),1:32 (**c**),until the disappearance of significant immunofluorescence at adilution of 1:64 (**d**). Scale bar: 40×.

To improve the sensitivity and specificity of this method, we considered the sera to be positive starting from a titer of 1:8 onwards and with immunostaining involving some but not all cells [21,22,23,24]. In particular, we considered sera positive for APA and AHA at low titer at a dilution of 1:8, at middle titer at dilution 1:16, and at high titer at dilution ≥ 1:32. APA and AHA were evaluated by two different operators in a double-blind (Figure 1 and Figure 2).

### 2.5. Progression-Free Survival

In our cross-sectional population, we assessed progression-free survival (PFS). PFS was estimated using theKaplan–Meiermethod. Kaplan-Meier curves compared PFS in the patients with non-small-cell lung cancer and melanoma with and without APA/AHA.

### 2.6. Statistical Analysis

Results are presented as mean and standard deviation. All the data passed the Kolmogorov–Smirnov normality test. Differences between frequencies were evaluated by the chi-squared or Fisher exact test. Differences between the two groups were evaluated by *t*-test. A value of *p* < 0.05 was considered to be statistically significant. All statistical analyses were performed using the SPSS 13.0 program (SPSS Inc., Chicago, IL, USA).

## 3. Results

Regarding the cross-sectional study, the characteristics of patients of Group 1 and controls are reported in Table 1.

The autoimmune pattern of those treated with anti-PD-1 (Group 1a) and those treated with anti-PD-L1 (Group 1b) are presented in Table 2. None of the controls were APA- or AHA-positive. Instead, a significant increase inthe prevalence of APA and AHA was observed in patients in Group 1. In particular, 26 out of 54 (48%) patients in this group had anti-pituitary or anti-hypothalamus antibodies at different titers: 21 of them (39%) had APA, 16 (29%) had AHA, and 11 (20%) had both AHA and APA. Moreover, a significant increase of TgAb and TPOAb titers was observed in Group 1 patients versus controls (Table 1).Considering the behavior related to the kind of treatment, we found a non-significant higher prevalence of APA and AHA in patients on anti-PD-L1 therapy (Table 2). In particular, 14 of 37 (38%) patients in Group 1a (anti-PD-1) were APA-positive, and eight were also AHA-positive; by contrast, 10 of 37 (27%) patients were positive only for AHA. Among patients treated with anti-PD-L1 antibodies (Group 1b), we found that 7 of 17 patients (41%) were APA-positive and three were also AHA-positive; in contrast, 6 of 17 (35%) patients were only positive for AHA (Table 2). Regarding the antibody titer, we found that among patients positive for APA in Group 1a, five were positive at low titer (1:8) and nine at medium titer (1:16), whereas among those positive for AHA, three were positive at low titer, seven at medium titer, and one at high titer (1:32); among patients in Group1b, all seven who were positive for APA were positive at medium titer, while among the six patients positive for AHA, five were positive at medium titer and one at high titer. One patient positive for APA/AHA developed adrenal insufficiency, with low levels of ACTH and cortisol and no history of corticosteroid treatments. Two patients positive for APA/AHA developed other endocrine toxicities, insulin-dependent diabetes mellitus, and primary hypothyroidism (Table 2).

We also evaluated the presence of autoimmunity in relation to the time from the start of immunotherapy, and we observed that 24 patients had been on treatment for less than 6 months and 30 for at least 6 months; 9 of 24 patients (37.5%) in treatment for less than 6 months were APA- or AHA-positive, while 17 of 30 patients (56%) who had been on treatment for at least 6 months were APA-or AHA-positive (*p* = 0.26). Evaluating the hormonal and biochemical findings of patients in Group 1, globally considered, we found higher FSH and LH, with lower testosterone concentrations in males and lower FSH levels in females, than in controls. Moreover, patients in Group 1 showed significantly lower sodium levels and diastolic arterial pressure than controls and a mild but significant increase of thyroid antibodies.

**Figure 3:**
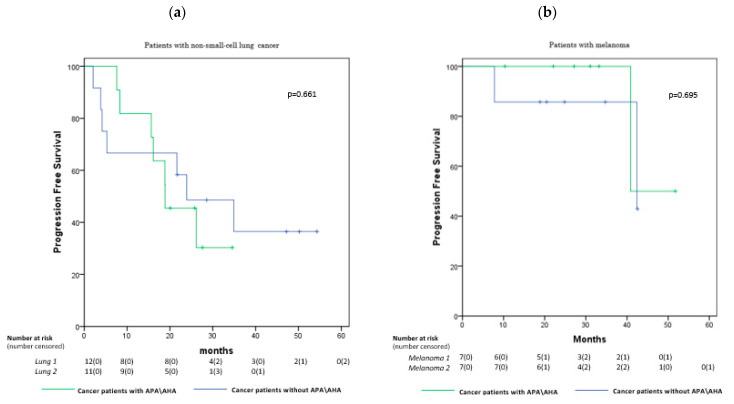
Kaplan–Meier curves comparing PFS in the patients with non-small-cell lung cancer (**a**) and melanoma (**b**) showing no significant differences in the groups with and without APA/AHA: 18.8 vs. 23.9 months, respectively, in patients with non-small-cell lung cancer (*p* = 0.661) and 40.9 vs. 42.4 months, respectively, in patients with melanoma (*p* = 0.695).

Finally, we found no significant difference in both melanoma and lung cancer patients in median overall progression-free survival (PFS) between those who were APA/AHA-positive and those who were APA/AHA-negative: 18.8 vs. 23.9 months in patients with lung cancer, respectively (*p* = 0.661), and 40.9 vs. 42.4 months in patients with melanoma, respectively (*p* = 0.695) (Figure 3a,b).

As regards the preliminary data of our longitudinal study, we found that only 1 of 13 patients (7.7%) in Group 2 was already APA-positive at the start of immunotherapy, whereas 7 of 13 (53%) became APA-positive and 3 (23%) AHA-positive after 9 weeks of treatment. Comparing biochemical and hormonal concentrations at baseline and at 9 weeks, we found a significant increase in glycemia and prolactin and a reduction in ACTH and IGF-1 levels at 9 weeks compared with basal values not attributable to drugs or intercurrent illnesses (Table 3). None of the patients enrolled forthe cross-sectional or longitudinal study developed any other immune-related adverse events along the time span of observation.

## 4. Discussion

The results of our cross-sectional study, aimed at assessing the prevalence of APA and, for the first time, AHA in cancer patients treated with anti-PD-1 and anti-PD-L1 antibodies, showed a statistically significant increase in the prevalence of AHA and APA in these patients compared with the control group. This high prevalence was confirmed by the preliminary data, even if related to a very short period of immunotherapy, of our longitudinal study, highlighting a possible pivotal role of these antibodies in evoking hypothalamic–pituitary autoimmunity in patients treated with these agents.

Several studies have been performed, seeking a possible link between cancer and autoimmune diseases. Their results seem to confirm this link since some autoimmune diseases occur more frequently in cancer patients, suggesting a possible cancer role in triggering autoimmunity; on the other hand, some autoimmune diseases may be predisposed to cancer [25,26]. Studies have so far reported that hypophysitis caused by ICIs occurs more commonly in cancer patients treated with ipilimumab (anti-CTLA4), where its prevalence is of about 12%, than in cancer patients receiving anti-PD-1 (nivolumab, pembrolizumab, cemiplimab) or anti-PD-L1 (atezolizumab, avelumab, durvalumab) treatment, in whom the reported prevalence is about 0.5% [7,8,9,10,11,12,13,14,15,19]. These patients instead had a higher prevalence of autoimmune and non-autoimmune thyroid diseases, especially hypothyroidism, but also hyperthyroidism and painless thyroiditis [7,8,9,10,16,17]. However, the prevalence of hypophysitis in patients treated with anti-PD-1 and anti-PD-L1 increases when these ICIs are associated with anti-CTLA-4 antibodies [7] or when patients have pre-existing autoimmune or inflammatory disorders [16]. The different prevalence of irAEs across ICIs has been attributed to their different mechanisms of action: antibodies blocking PD-1/PD-L1 seem to act by enhancing a pre-existing CD8 T-cell response, whereas those blocking CTLA-4 act by enhancing T-cell priming [27]. The increased prevalence of hypophysitis in patients treated with anti-CTLA-4 has also been related to a possible ectopic expression of CTLA-4 at the pituitary level, which may act as an autoantigen evoking an autoimmune attack by anti-CTLA-4 antibodies when administered in cancer patients [6,28]. Finally, an increase of T-cell activity against antigens shared by tumors and normal tissues, evoked by an increase of pre-existing antibodies or inflammatory cytokines, has been suggested as a further mechanism [29]. Lymphocytic hypophysitis (LYH) is the most frequent form of primary hypophysitis, and it is characterized by an extensive infiltration of the anterior pituitary by lymphocytes and plasma cells, which can also involve the posterior pituitary and infundibulum [30,31,32,33,34]. Several studies have investigated the role of anti-pituitary and anti-hypothalamus antibodies in LYH [20,21,31,32,33,34]; however, studies on the detection of these antibodies in irAEs, occurring in cancer patients treated with ICIs and especially in those treated with PD-1 and PD-L1 inhibitors, are rare and have only addressed the occurrence of APA but not of AHA. In particular, APA hasrecently been detected in two out of four cancer patients presenting with hypophysitis related to anti-PD-L1 or anti-PD-1 treatment [19]. Clinical and hormonal findings of the four patients, association with other irAEs, and the grade of severity of hypophysitis were extremely variable in these patients [18]. However, central hypoadrenalism and hyponatremia were constantly detected, even if sellar MRIs did not reveal significant findings of pituitary inflammation [19].

The persistence of normal basal function in our APA/AHA-positive patients may be due to the short time span of treatment, and they could develop a pituitary dysfunction overa more prolonged time of treatment. Concerning this, the presence of lower sodium levels and lower diastolic pressure could indicate possible incoming secondary hypoadrenalism. This also takes into account the results from a previous longitudinal study that showed that the presence of AHA/APA at high titer in patients with still normal pituitary function may represent a sensitive marker for the development of future pituitary dysfunction [20,33,35]. In this connection, even if our protocol does not allow us to fix the timing of IRAE-related hypophysitis in our patients who are positive for AHA/APA but still with normal pituitary function, we think it is advisable to treat these patients, especially when positive at high titer, with appropriate corticosteroid therapy in order to attempt to interrupt the progression from potential to subclinical and, finally, to clinically overt diseases.

Other interesting findings emerging from our results are that our male patients in Group 1, positive for APA/AHA, showed higher LH and FSH levels but lower testosterone levels than controls, suggesting primary hypogonadism, while female patients had only a significant reduction in FSH concentrations. The lack of data on testicular antibodies in our patients does not allow the drawing of a conclusion on the possible involvement of testicular autoimmunity evoked by anti-PD-L1/PD-1 therapy. However, possible direct iatrogenic gonadal damage caused by this therapy or by previous therapies may not be excluded. On the contrary, the reduction of FSH in menopausal patients may suggest a pituitary involvement affecting FSH secretion by gonadotrophs.

To clarify these aspects, it will be helpful to complete the planned longitudinal study, integrating it with dynamic tests of pituitary and satellite gland function at the next observations until the end of the study in order to also disclose possible subclinical alterations. Moreover, the prolonged longitudinal observation may allow us to verify whether some AHA/APA-positive patients will develop other immune-related adverse events. The preliminary results of our longitudinal study show a significant reduction of ACTH and IGF-1 concentrations and a significant increase of prolactin after 9 weeks of immunotherapy with respect to the baseline, suggesting a precocious autoimmune pituitary involvement.

The detection for the first time of AHA but not APA in some of our patients suggests that a hypothalamic autoimmune process may be responsible in some cases for secondary pituitary dysfunction or may contribute to pituitary dysfunction in patients in whom they are associated with APA. Clinical aspects of autoimmune hypothalamitis occurring in five patients have been recently described by Wei Q. et al. [36]. All patients presented with diabetes insipidus, headache, hypopituitarism, hyperprolactinemia, and hypothalamic syndrome. The authors considered that this form was a subtype of autoimmune hypophysitis based onhistopathological and RMI findings and the comorbidity with other autoimmune diseases in some of the five patients. However, in none of them did they look for anti-pituitary or anti-hypothalamic antibodies [36]. The possible occurrence of hypothalamitis in patients treated with these ICIs has been recently reported [37], and it may be favored by the demonstrated expression of PD-L1 at the hypothalamic level, which can mediate hypothalamitis [38]. However, in these cases, data on the antibody pattern were also lacking. More recently, a paper by Ture et al., in collaboration with our lab, investigated the autoantibody pattern in a 35-year-old-woman with autoimmune hypopituitarism and central diabetes insipidus. They found the presence of anti-hypothalamus antibodies to AVP-secreting cells at high titer in the absence of anti-pituitary antibodies, suggesting the diagnosis of isolated autoimmune hypothalamitis. The authors, also reviewing the literature on this issue, concluded by affirming that hypothalamitis may be considered a novel autoimmune endocrine disease [39]. None of our AHA-positive patients in treatment with anti-PD-1/PD-L1 antibodies showed central diabetes insipidus. Thus, it may be hypothesized that in these patients, the AHAs were directed towards hypothalamic-releasing hormone-secreting cells rather than to AVP-secreting cells, thus causing the secondary impairment of pituitary secretions rather than diabetes insipidus. This assumption has to be confirmed by further studies bytesting the sera with double four-layer immunofluorescence in order to identify the hypothalamic cells targeted by AHA.

One patient positive for APA and AHA developed insulin-dependent diabetes mellitus related to PD-1 inhibitor therapy. In fact, treatment with PD-1/PD-L1 inhibitors may also lead to the onset of other endocrinopathies. Other than the more frequently occurring thyroid disorders, diabetes mellitus and primary adrenal insufficiency can occur during immunotherapy but more rarely [40,41,42,43,44].

Finally, no significant differences in survival, expressed as PFS, were observed between the groups of patients with and without APA/AHA, likely due to the limited time span from the start of the therapy. This result needs to be confirmed in the longitudinal study.

The three main limitations of our study were:

- The patients were included in the study at different times from the start of immunotherapy and in a highly variable time span, ranging from 4 weeks to 34 months of therapy;
- No dynamic tests were performed to assess the presence of subclinical pituitary dysfunction;
- Lack of characterization by four-layer double immunofluorescence of hypothalamic–pituitary-secreting cells targeted by these antibodies, which has now been planned for the next steps of our longitudinal study.

Nevertheless, the significantly increased prevalence of APA/AHA found in patients included in the cross-sectional study provides us with some interesting data that need to be clarified with further longitudinal studies.

However, the preliminary data of our longitudinal study, already showing a significantly higher prevalence of APA and a non-significant higher prevalence of AHA in patients treated with anti-PD-1 antibodies after 9 weeks, seem to confirm the results of the observational cross-sectional study. In particular, the detection, for the first time, of AHA but not APA in some of our patients seems to confirm that a hypothalamic autoimmune process is responsible, in some cases, for secondary pituitary dysfunction in patients treated with ICIs. The completion of our longitudinal study will allow us, at the end of the second year of observation, to evaluate the timing of appearance of APA/AHA and to correlate them to the development of pituitary dysfunction by periodically examining the results of hormonal and more complete immunological findings of the enrolled patients. Finally, we may be able to assess whether the presence of pituitary/hypothalamic autoimmunity impinges the prognosis of the malignancies.

## 5. Conclusions

The prevalence of APA and AHA during anti-PD-1/anti PD-L1 therapy is higher than in healthy people, and some patients may already develop APA/AHA positivity after 9 weeks of immunotherapy. The planned longitudinal study and other studies will better clarify the role of these antibodies as predictive of pituitary deficiencies and if they may predict other endocrine toxicities and the cancer prognosis.

## Figures and Tables

**Table 1:** Clinical, biochemical, and hormonal values in patients on treatment with anti-PD-1, anti-PD-L1, or both (Group 1) and controls. Values are mean ± SD.

	Patients on Treatment with Anti-PD-1, Anti-PD-L1 or Both (Group 1, *n* = 54)	Control Group (*n* = 50)	*p*-Value
Sex (% of male)	65	59	0.76
Age, y	65 ± 10.7	67 ± 8	0.28
Systolic Arterial Pressure, mm Hg	119 ± 15	122 ± 12	0.26
Diastolic Arterial Pressure, mm Hg	72 ± 11	78 ± 10	0.005
Sodium, mmol/L	139 ± 3	142 ± 3	<0.001
Glycemia, mg/dL	103 ± 24	95 ± 14	0.04
ACTH, pg/mL	21 ± 17	22.7 ± 3.6	0.49
Cortisol, μg/dL	13.8 ± 6.8	13.4 ± 1.5	0.68
TSH, μUI/mL	1.7 ± 1.6	1.5 ± 0.4	0.39
FT4, ng/dL	1.27 ± 0.67	1.2 ± 1.8	0.79
FT3, pg/mL	3.4 ± 1.8	3.7 ± 0.6	0.26
TgAb, UI/mL	25.4 ± 15.3	8.06 ± 3.70	<0.001
TPOAb, UI/mL	28.1 ± 39.03	6.30 ± 8.18	<0.001
FSH, UI/L			
Males	26.3 ± 18.29	3.2 ± 1.8	<0.001
Females	61.2 ± 39.8	81.7 ± 34.5	0.006
LH, UI/L			
Males	7.87 ± 5.38	3.8 ± 1.7	<0.001
Females	18.4 ± 12.02	21.9 ± 9.4	0.10
Oestradiol, pg/mL (females)	16.5 ± 12.1	12.7 ± 8.3	0.06
Testosterone, ng/dL (males)	209.2 ± 159.31	312.1 ± 172.4	0.002
IGF-1, ng/mL	122.3 ± 45.3	134.5 ± 40.2	0.15
Prolactin, ng/mL	14.7 ± 5.59	18.6 ± 14.2	0.06
Patients positive for APA, n (%)	21 (39%)	0	<0.001
Patients positive for AHA, n (%)	16 (29%)	0	<0.001
Patients positive for APA and AHA, n (%)	11 (20%)	0	0.002

**Table 2:** Detection of anti-pituitary (APA) and anti-hypothalamus (AHA) antibodies in patients on anti-PD-1 (Group 1a) and anti-PD-L1 (Group 1b) therapy. Values are mean ± SD.

	Patients on Anti-PD-1 Therapy (Group 1a) (*n* = 37)	Patients on Anti-PD-L1 Therapy (Group 1b) (*n* = 17)	*p*-Value
Time from the start of therapy, month	8.9 ± 8.8	6.8 ± 8.1	0.40
Patients with APA positivity, n (%)	14 (38%)	7 (41%)	0.94
Low titer 1:8	5 (13.5%)	0	0.16
Middle titer 1:16	9 (24.3%)	7 (41%)	0.14
High titer 1:32	0	0	
Patients with AHA positivity, n (%)	10 (27%)	6 (35%)	0.76
Low titer 1:8	3 (8%)	0	0.54
Middle titer 1:16	7 (19%)	5 (29%)	0.48
High titer 1:32	1 (2.7%)	1 (6%)	0.53
Patients positive for APA/AHA and pituitary deficiency, n (%)	1 (2.7%)	0	0.99
Patients positive for APA/AHA and other endocrine toxicities, n (%)	2 (5.4%)	0	0.99

**Table 3:** Biochemical, hormonal, and autoimmune profiles of 13 patients (Group 2) before and after 9 weeks of therapy with ICIs. Values are mean ± SD.

	Before Treatment with ICIs	After 9 Weeks on Therapy with ICIs	*p*-Value
Systolic arterial pressure, mm Hg	136 ± 15	130 ± 20	0.05
Diastolic arterial pressure, mm Hg	88 ± 12	80 ± 16	0.16
Glycemia, mg/dL	90 ± 9	98 ± 10	0.04
ACTH, pg/mL	25 ± 7	17 ± 9	0.01
Cortisol, μg/dL	10.8 ± 5.39	10.1 ± 4.49	0.72
TSH, μUI/ mL	2.5 ± 1.7	2.3 ± 1.4	0.74
FT4, ng/dL	1.5 ± 0.8	1.1 ± 0.9	0.24
FT3, pg/mL	3.4 ± 0.8	2.9 ± 1	0.17
TgAb, UI/mL	24.3 ± 52.05	115.7 ± 356.3	0.36
TPOAb, UI/mL	50.06 ± 107.7	39.7 ± 85.5	0.78
FSH, mUI/mL			
males	22.1 ± 18.25	24.9 ± 19.5	0.69
females	79.1 ± 31.5	68.7 ± 53.4	0.55
LH, mUI/mL			
males	10.7 ± 8.48	7.64 ± 5.64	0.28
females	21.3 ± 15.7	20.8 ± 16.1	0.93
Oestradiol, pg/mL (females)	10.2 ± 1.28	14.2 ± 7.6	0.07
Testosterone, ng/dl (males)	162.5 ± 85.5	156.8 ± 84.4	0.86
IGF-1, ng/mL	148.3 ± 53.6	90.3 ± 35.0	0.003
Prolactin, ng/mL	6.6 ± 3.2	16.3 ± 15.8	0.04
Patients positive for APA, N (%)	1 (7.7%)	7 (53%)	0.03
Patients positive for AHA, N (%)	0	3 (23%)	0.22

## Data Availability

All data generated or analyzed during this study are included in this published article or in the data repositories listed in the references.
